# Giant Cell Arteritis With Sepsis-Like Symptoms in an Elderly Man: A Case Report

**DOI:** 10.7759/cureus.61074

**Published:** 2024-05-25

**Authors:** Hisae Minamioka, Yuto Tsukihashi, Tasuku Yano, Chiaki Sano, Ryuichi Ohta

**Affiliations:** 1 Family Medicine, Shimane University Medical School, Izumo, JPN; 2 Community Medicine Management, Shimane University Faculty of Medicine, Izumo, JPN; 3 Community Care, Unnan City Hospital, Unnan, JPN

**Keywords:** rural, family medicine, general medicine, geriatrics, glucocorticoids, diagnostic errors, sepsis, vasculitis, giant cell arteritis

## Abstract

This case report details the diagnostic challenge and management of an 88-year-old man who presented to a rural Japanese community hospital with sepsis-like symptoms, initially suspected of acute bacterial cholangitis based on his physical and laboratory findings. Despite the antibiotic treatment of tazobactam and piperacillin, the patient's symptoms persisted, leading to further investigations that revealed no signs of infection but notable aortic arch wall thickening on contrast-enhanced computed tomography scans. These findings, combined with the patient's clinical presentation and lack of antibiotic response, redirected the diagnosis toward giant cell arteritis (GCA). The administration of prednisolone of 60 mg daily significantly alleviated symptoms and prevented potential severe complications such as blindness and irreversible neurological damage. This case underscores the importance of considering GCA in elderly patients presenting with systemic inflammatory symptoms and the necessity of timely intervention. It also highlights the challenges in managing high-dose steroid therapy in elderly patients and suggests the potential benefits of integrating immunosuppressants to reduce steroid dependency. This report emphasizes the need for heightened awareness and a comprehensive diagnostic approach in atypical presentations of GCA, particularly in geriatric populations within resource-limited healthcare settings.

## Introduction

Giant cell arteritis (GCA) is a vasculitis that often affects large to medium-sized arteries and occurs in elderly people [1]. It is one of the differential diagnoses of fever of unknown origin. The age of onset is usually 50 years and over, with a peak in the 60s and 70s and an annual incidence of 10 in 100,000 adults over 50 years old [2]. GCA is slightly more common in women [2]. The chief complaints seen in the early stages are nonspecific, such as fever, general malaise, neck pain or pain in various parts, and dizziness, with symptoms similar to initial presentations of multiple infections [3,4]. GCA occurs mainly in the elderly and shows various unspecific symptoms, often requiring a comprehensive diagnostic process from diagnosis to treatment [2,5].

Our case had symptoms similar to acute bacterial infection and was ultimately diagnosed as GCA. The patient's initial symptoms suggest bacterial infection and cholangitis, such as shaking chill, right upper quadrant pain, shock vital signs, and liver tenderness on percussion. Through subsequent tests, we could diagnose and treat giant cell arteritis at an early stage. Early intervention in giant cell arteritis can prevent blindness and irreversible neurological damage. Through this case, we discuss the diagnosis and treatment strategies for GCA in a rural community hospital.

## Case presentation

An 88-year-old man visited a rural community hospital with a chief complaint of difficulty moving. The patient had been independent and had no symptoms until the day before admission. On the day of admission, when he tried to go to the bathroom after waking up, he became aware of the weakness in his lower limbs. He had difficulty moving and was transported to our hospital for emergency care. There were no respiratory and abdominal symptoms and no apparent joint pain. He had no travel history and had no contact with infected people. He had no history of keeping pets. He has a medical history of type 2 diabetes, hyperlipidemia, hypertension, folic acid deficiency, low back pain, and chronic bronchitis. The drug history includes vildagliptin 100 mg daily, metformin 1000 mg daily, tizanidine 2 mg daily, bezafibrate 200 mg daily, and gliclazide 40 mg daily.

On the initial arrival, his level of consciousness was clear, and his vital signs were blood pressure 135/71 mmHg, pulse 108 beats/min, respiration 21 breaths/min, body temperature 37.9°C, and SpO_2_ 97% (room air). Physical examination revealed mild pallor and icterus of the ocular conjunctiva without petechial hemorrhages. A diastolic murmur was heard without abnormal lung sounds. Right hypochondrium tenderness and liver tenderness were present on the percussion in the abdomen. He had no abnormal neurological findings. Blood tests revealed elevated hepatobiliary enzymes, hyperglycemia, white blood cells, and inflammatory markers, such as C-reactive protein and erythrocyte sedimentation rate (Table 1).

**Table 1:** Initial laboratory data of the patient. CRP: C-reactive protein; Ig: immunoglobulin.

Parameter	Level	Reference
White blood cells	12.70	3.5–9.1 × 10^3^/μL
Neutrophils	84.3	44.0–72.0%
Lymphocytes	19.5	18.0–59.0%
Hemoglobin	10.3	11.3–15.2 g/dL
Hematocrit	29.5	33.4–44.9%
Mean corpuscular volume	95.6	79.0–100.0 fl
Platelets	34.7	13.0–36.9 × 10^4^/μL
Erythrocyte sedimentation rate	124	2–10 mm/hour
Total protein	8.1	6.5–8.3 g/dL
Albumin	3.3	3.8–5.3 g/dL
Total bilirubin	1.8	0.2–1.2 mg/dL
Direct bilirubin	1.2	0.0-0.4 mg/dL
Aspartate aminotransferase	43	8–38 IU/L
Alanine aminotransferase	22	4–43 IU/L
Alkaline phosphatase	274	38-113 U/L
Lactate dehydrogenase	213	121–245 U/L
Blood urea nitrogen	22.2	8–20 mg/dL
Creatinine	0.92	0.40–1.10 mg/dL
Serum, Na	134	135–150 mEq/L
Serum, K	3.7	3.5–5.3 mEq/L
Serum, Cl	97	98–110 mEq/L
Ferritin	625.2	14.4–303.7 ng/mL
CRP	33.71	<0.30 mg/dL
IgG	1729	870–1700 mg/dL
IgM	97	35–220 mg/dL
IgA	343	110–410 mg/dL
Urine test	-	-
Leukocyte	Negative	Negative
Protein	Negative	Negative
Blood	Negative	Negative

**Figure 1:**
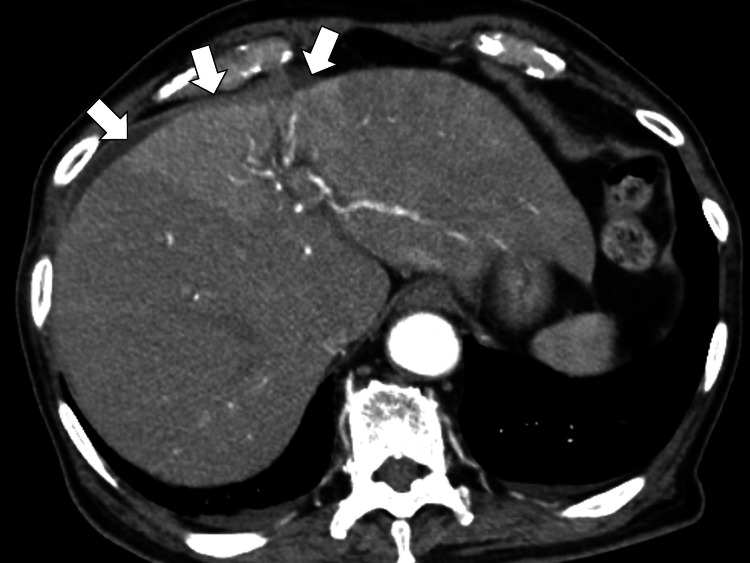
Contrast-enhanced computed tomography of the abdomen revealing a partial enhancement of liver and intrahepatic bile ducts (white arrows).

Abdominal computed tomography (CT) and magnetic resonance imaging (MRI) showed no significant bile duct dilatation or common bile duct stones. A contrast-enhanced abdomen CT revealed a partial enhancement of the liver and intrahepatic bile ducts (Figure 1).

His brain MRI did not show any abnormality, ruling out the possibility of a brain stroke. He was diagnosed with acute bacterial cholangitis from the physical, laboratory, and CT findings and treated with 2 g of cefmetazole intravenously.

**Figure 2:**
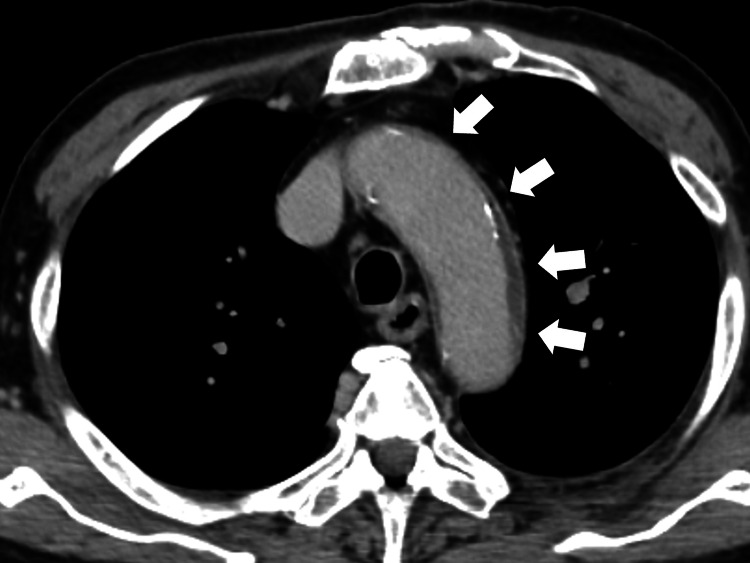
Chest-to-pelvic contrast-enhanced computed tomography showing wall thickening of the aortic arch (white arrows).

On the eighth day of hospitalization, fever persisted, and cefmetazole was changed to tazobactam/piperacillin of 13.5/day g, considering the possibility of hospital-acquired infection such as *Pseudomonas aeruginosa* infection. However, the fever continued, and contrast-enhanced CT showed no specific findings of cholangitis or cholecystitis, but wall thickening of the aortic arch was observed (Figure 2).

On the 10th day of hospitalization, the possibility of infection was ruled out because of negative blood, urinary, and sputum cultures, and the patient's stable vital signs except for fever. The additional tests for antinuclear antibodies, rheumatoid factors, anti-citrullinated protein antibodies, and anti-neutrophil cytoplasmic antibodies were negative. Based on the contrast-enhanced CT findings, the possibility of GCA was considered. 

On the 10th day of hospitalization, as the fever continued, prednisolone of 60 mg/day was started for the diagnosis of GCA based on the guideline [1]. Oral administration of sulfamethoxazole (400 mg)/trimethoprim (80 mg) was started to prevent *Pneumocystis pneumonia*. On the 13th day of hospitalization, 50 mg of minodronic acid was administered orally to prevent osteoporosis. The dose of prednisolone was continued to be reduced from 5 to 10 mg/two weeks by monitoring the symptoms and fever. Through the discussion with a patient and families, as a sparing drug for prednisolone, methotrexate (MTX) was chosen because of the cost of biologics such as tocilizumab. The dose of MTX was increased to 16 mg/week. In the process of the treatment, he had hypoactive delirium and hyperglycemia because of prednisolone usage. He was rehabilitated intensively and treated with insulin for glucose control. He was transferred to the rehabilitation unit for the discharge to home.

## Discussion

In this study, we experienced a case of giant cell arteritis that presented with sepsis-like symptoms and acute onset of fever, malaise, and lower leg weakness. Giant cell arteritis develops in old age, has an acute onset, and can cause multiorgan damage due to systemic inflammation, making it difficult to differentiate from sepsis in its early stages by quickly making an appropriate diagnosis of exclusion, early diagnosis, and early treatment become possible, which may improve the quality of life of patients with giant cell arteritis.

It can be challenging to differentiate giant cell arteritis from sepsis in the early stages. In this case, abdominal contrast-enhanced CT showed a contrast effect in the liver, so bacterial cholangitis was suspected, and treatment with cefmetazole was performed. However, the patient showed no response to the treatment [6]. Next, considering hospital-acquired infections, such as *Pseudomonas aeruginosa*, the patient was changed to tazobactam/piperacillin, but no response was observed. Blood culture results were also negative, and the possibility of infection was ruled out [7]. The condition was progressive, and contrast-enhanced CT findings showed thickening of the wall of the aortic arch, so treatment with prednisolone was started, considering the possibility of giant cell arteritis.

Glucocorticoids are effective for giant cell arteritis and are the first-choice drug. This case also experienced remission of clinical symptoms with appropriate treatment. This could reduce the risk of blindness and irreversible neurological damage, which increases with delayed treatment [8]. On the other hand, high-dose steroid therapy for elderly patients may cause various complications. It has been pointed out that, as in this case, prolonged delirium and hyperglycemic conditions may worsen the prognosis [9]. Although this was not possible in this case, it may be practical to introduce a biological agent early and quickly reduce the amount of steroid used [10].

In this case, we believe a comprehensive thought process is necessary for treating autoimmune diseases in the elderly. General medicine at rural hospitals has an increasing number of opportunities to treat various patients with fevers [11]. Among these patients, there are patients with vasculitis who come to rural hospitals with sepsis-like symptoms, such as the one in this case [12]. In particular, diseases such as giant cell arteritis, whose primary pathophysiology is innate immunity, can cause irreversible changes if they develop acutely and are not treated smoothly [13]. As general physicians, while always taking an appropriate approach to sepsis, it is necessary to reconsider the diagnosis if there is a poor response to treatment within a few days and the progression is not typical of sepsis [14]. Family physicians need to change their thinking to consider other pathological conditions centered on innate immunity [15].

## Conclusions

This case emphasizes the diagnostic complexity of GCA in elderly patients presenting with symptoms mimicking sepsis. Initially misdiagnosed as bacterial cholangitis, persistent symptoms, and unresponsiveness to antibiotics necessitated further evaluation, leading to a GCA diagnosis. Early intervention with prednisolone significantly improved the patient's condition, underscoring the importance of timely diagnosis and treatment in preventing severe complications like blindness. This case highlights the necessity for a broad differential diagnosis and consideration of autoimmune conditions in elderly patients presenting with atypical symptoms to improve outcomes and minimize unnecessary treatments.
